# Corneal Biomechanics Determination in Healthy Myopic Subjects

**DOI:** 10.1155/2016/2793516

**Published:** 2016-07-21

**Authors:** Kunliang Qiu, Xuehui Lu, Riping Zhang, Geng Wang, Mingzhi Zhang

**Affiliations:** Joint Shantou International Eye Center of Shantou University and the Chinese University of Hong Kong, Shantou, Guangdong Province 515041, China

## Abstract

*Purpose*. To determine the corneal biomechanical properties by using the Ocular Response Analyzer*™* and to investigate potential factors associated with the corneal biomechanics in healthy myopic subjects.* Methods*. 135 eyes from 135 healthy myopic subjects were included in this cross-sectional observational study. Cornea hysteresis (CH), corneal resistance factor (CRF), cornea-compensated intraocular pressure (IOPcc), and Goldmann-correlated intraocular pressure (IOPg) were determined with the Reichert Ocular Response Analyzer (ORA). Univariate and multivariate regression analyses were performed to investigate factors associated with corneal biomechanics.* Results*. The mean CH and CRF were 9.82 ± 1.34 mmHg and 9.64 ± 1.57 mmHg, respectively. In univariate regression analysis, CH was significantly correlated with axial length, refraction, central corneal thickness (CCT), and IOPg (*r* = −0.27, 0.23, 0.45, and 0.21, resp.; all with *p* ≤ 0.015), but not with corneal curvature or age; CRF was significantly correlated with CCT and IOPg (*r* = 0.52 and 0.70, resp.; all with *p* < 0.001), but not with axial length/refraction, corneal curvature, or age. In multivariate regression analysis, axial length, IOPcc, and CCT were found to be independently associated with CH, while CCT and IOPg were associated with CRF.* Conclusions*. Both CH and CRF were positively correlated with CCT. Lower CH but not CRF was associated with increasing degree of myopia. Evaluation of corneal biomechanical properties should take CCT and myopic status into consideration.

## 1. Introduction

Myopia is a worldwide common ocular disorder and the prevalence rate of myopia was reported to be 26.7% and 26.2% in the Handan Eye Study and the Beaver Dam Study, respectively [1–3]. During the past decades, laser in situ keratomileusis (LASIK) has been developed as one of the most important techniques of refractive correction in myopic eyes. However, there have been reports of keratectasia developed after LASIK, possibly resulting from thickness and biomechanical changes of the cornea [4–6]. Understanding of the corneal biomechanical properties in myopic eyes might help refractive surgeons identify eyes at risk of developing keratectasia after refractive surgery. Although measurement of corneal thickness has been proposed to be a useful parameter for the clinical identification of keratoconus [7, 8], our knowledge about corneal properties is far from complete.

Recently, the Ocular Response Analyzer (ORA, Reichert Ophthalmic Instruments, Depew, NY, USA) has been developed to measure* in vivo* corneal biomechanical properties [9, 10], which has been reported to be useful in the differentiation of healthy and diseased corneas [11–14]. However, variations of corneal biomechanical properties, which may confound the detection of cornea disease, have been shown in healthy subjects [15–20]. And conflicting data regarding the relationship between myopia and corneal hysteresis has been reported in previous studies [21–27]. While some studies show that lower CH was associated with longer axial length [21–24], a number of studies reported that no significant relationship between myopia status and CH was detected [25–27]. Thus, controversies exist regarding the association between corneal biomechanical properties and myopic status.

In view of the clinical importance of corneal hysteresis and the controversies about the relationship between corneal hysteresis and myopic status, we aimed to investigate the potential factors that affect the corneal biomechanics in healthy myopic eyes.

## 2. Materials and Methods

### 2.1. Subjects

One hundred and forty-six Chinese healthy myopic subjects with a spherical equivalent less than −0.5 diopters (D) were consecutively recruited from the Refractive Surgery Clinic of Joint Shantou International Eye Center. One eye from each subject was randomly selected. All the included subjects received a full ophthalmic examination including the measurement of visual acuity, refraction, central corneal thickness (A-ultrasound pachymeter; Reichert Ophthalmic Instruments, Depew, NY, USA), axial length (IOLmaster; Carl Zeiss Meditec, Dublin, CA), and a dilated fundus stereoscopic examination. None of the included eyes had any concurrent ocular disease other than a refractive error. Subjects with best corrected visual acuity less than 20/20, intraocular pressure over 21 mmHg, positive family history of glaucoma, contact lens used, ocular surgery, glaucoma, or diabetes were excluded. The study was conducted in accordance with the ethical standards stated in the Declaration of Helsinki and approved by the local clinical research ethics committee. Written informed consent was obtained from each subject before enrollment.

### 2.2. Ocular Response Analyzer Measurement

All the included eyes received measurements of corneal biomechanical properties by using ORA. The principle of ORA has been described elsewhere [9, 10]. In brief, ORA works by pushing a precisely metered collimated-air-pulse onto the surface of the cornea. It causes the cornea to move inwards, past applanation, and into a slight concavity and then return to its normal configuration after the air pump shuts off and as the pressure decreases. During the entire 20-millisecond measurement process, two independent pressure values (P1 and P2) derived from the inward and outward applanation events are recorded. Four main parameters including corneal hysteresis (CH), corneal resistance factor (CRF), cornea-compensated intraocular pressure (IOPcc), and Goldmann-correlated intraocular pressure (IOPg) are provided by the device. According to the manufacturer, CH is thought to be the quantitative measurement of viscous damping in the corneal tissue while CRF is a measurement of the cumulative effects of both the viscous and elastic resistance.

To ensure accurate measurement, the ORA software (Software Version 2.02, Reichert Ophthalmic Instruments, Depew, NY, USA.) provides a quality check score (waveform score, ranging from 0 to 10) based on the measurement curves. A score of 5 was set to be the minimum standard in this study. For each included eye, the ORA examination was performed at least 3 times. Disqualified measurements (the waveform score less than 5) and irreproducible values were discarded and retaken. The average values of three measurements with desirable curves were recorded for data analysis.

### 2.3. Statistics Analyses

Statistical analyses were performed with SPSS software version 13.0 (SPSS Inc., Chicago, IL). One-Sample Kolmogorov-Smirnov Test was used to evaluate if each parameter had a normal distribution. Univariate and multivariate regression analyses were performed to determine the effects of axial length/refractive error, cornea curvature, age, IOP, and CCT on the measurements of corneal biomechanics. A *p* value less than 0.05 was considered statistically significant.

## 3. Results

Eleven subjects were excluded because of repeatable visual field defects (4) and unacceptable ORA measurements (7). As a result, 135 eyes from 135 subjects were finally included in the analysis. The mean age, axial length, and spherical equivalent were 23.25 ± 4.58 years (range, 18 to 40), 25.63 ± 1.07 mm (range, 23.25 to 29.45), and −5.05 ± −2.02 D (range, −1 to −13.25 D), respectively. The corneal biomechanical characteristics of the study population were presented in Table 1. All parameters included in the univariate regression analysis were found to have a normal distribution (One-Sample Kolmogorov-Smirnov normality test, all with *p* ≥ 0.59).

**Figure 1:**
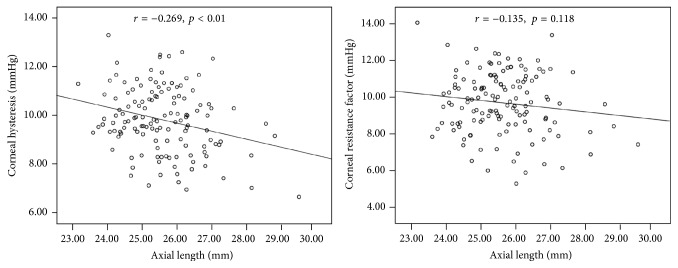
Scatter plots showing the correlation between corneal hysteresis, corneal resistance factor, and axial length.

**Figure 2:**
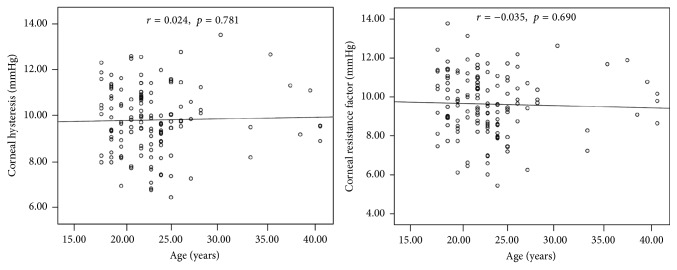
Scatter plots showing the correlation between corneal hysteresis, corneal resistance factor, and age.

Table 2 demonstrates the associations between corneal biomechanics and the potential factors. Axial length, spherical equivalent, CCT, and IOPcc were significantly correlated with CH (all with *p* ≤ 0.006). No significant relationship between corneal curvature, age, and CH was detected. Both IOPcc and CCT were significantly correlated with CRF (*r* = 0.203, *p* = 0.018 and *r* = 0.521, *p* < 0.001, resp.). CRF did not vary significantly with age, axial length, or spherical equivalent.  Figure 1 shows the association between CH, CRF, and axial length.  Figure 2 presents the relationship between CH, CRF, and age.

In the multivariate analysis, axial length, CCT, and IOPcc were all independently associated with CH (Table 3). Mean CH decreased by approximately 0.23 mmHg (*p* = 0.014) for every 1 mm greater axial length and by approximately 0.24 mmHg (*p* < 0.001) for every 10 *μ*m thinner CCT. Both CCT and IOPcc were independently and significantly associated with CRF (Table 4).

## 4. Discussion

In the present study, we assessed the determinants of corneal biomechanics in healthy myopic subjects. Both CH and CRF were significantly associated with CCT. We found that CH but not CRF and CCT decreased with increasing degree of myopia. By using multivariate analysis, our results indicated that decreased CH was significantly associated with increasing degree of myopia and thinner CCT independent of IOP level.

Associations between CH, CRF, and CCT have been investigated [15–17, 19, 20]. Significant and positive relationships between CH, CRF, and CCT were reported in most of the previous studies [15–17, 19, 20]. In concordance with previous reports, we found that CCT was significantly associated with both CH and CRF. Moreover, CCT was found to be the most prominent predictor for variations of CH and CRF. As CH and CRF are thought to be the measurements of viscous damping and overall elastic resistance of the cornea, one would not feel surprised to find significant associations between CH, CRF, and CCT. The current results indicate that evaluation of CH and CRF should be interpreted in light of CCT.

Previous studies have reported the association between CH and refractive status in healthy subjects [18–33]. Although no significant correlation was detected between CH and refractive error in some of the previous studies [25–28], most of the studies reported that CH was significantly associated with refractive error or axial length [18–24, 29–33]. Song et al. [21] reported that longer axial length was significantly associated with lower corneal hysteresis in Chinese children. In another study by Shen et al. [22], refractive errors of spherical equivalent were found to be significantly correlated with CH in 135 normal Chinese adults. In 293 healthy Spanish children, Bueno-Gimeno et al. [23] found that lower levels of CH were associated with longer axial length. In a recent study, a negative and weak correlation between axial length and CH was reported in 312 eyes of 177 Spanish healthy subjects [24]. In a population-based study, Narayanaswamy et al. reported that CH was significantly influenced by axial length in 1136 Chinese adults [20]. Consistent with most of the previous studies, we found a significant relationship between CH and axial length/refractive error in 135 Chinese myopic subjects.

In the present study, CH was significantly correlated with axial length/refraction, CCT, and IOPcc in the univariate analysis. Thus, analyzing these factors with multivariate analysis is crucial in determining their relative effects on measurement of CH. By using the multivariate regression analysis, we found that axial length, CCT, and IOPcc were significantly associated with CH. Our finding suggests that decreased CH is independently associated with increasing degree of myopia. The decrease of CH with increased axial length could be explained by the stretch of the periphery sclera observed in myopic eyes [34, 35]. Due to elongation of the globe, the cornea is also stretched which may result in decrease of viscoelastic properties observed in the present study. Further studies are warranted to investigate the underlying mechanism for the association between myopia and CH.

Decrease of CH and CRF has been observed in eyes after photorefractive keratectomy (PRK) and LASIK [11, 36, 37]. Moreover, the amount of biomechanical change was found to be associated with the amount of myopic correction and different flap creation techniques [38]. Although the clinical significance of alteration in corneal biomechanics has not been well studied, previous studies have shown that keratoconic eyes had significantly lower CH compared with control eyes [11, 39–41]. These results indicate that assessment of corneal biomechanics may provide additional information on keratoconus screening and grading. Previous studies have already reported the usefulness of corneal biomechanics in the differentiation of healthy and diseased corneas [11–14, 42]. In the present study, as decreased CH was found to be associated with increasing degree of myopia, evaluation of corneal biomechanics in corneal disorders should take axial length into consideration.

Interestingly, axial length was found to be associated with CH but not with CRF in the current study. According to the manufacturer, CH is a measurement of viscous damping in the corneal tissue while CRF is a measurement of the overall elastic resistance. As CH and CRF are both generated from P1 and P2 (CH = P1 − P2, CRF = P1 − 0.7 *∗* P2) [15, 16], they are both expected to be influenced by viscoelastic changes. However, our findings in the present study indicated that the change of CH may be independent of CRF. Although the exact underlying mechanism for the present finding is not clear, our results were similar to findings of previous studies [19, 22, 31]. Shen et al. reported that CH but not CRF was significantly correlated with refractive errors in 135 normal Chinese adults [22]. In a recent study, Wong and Lam also reported that axial length was significantly associated with CH but not with CRF [31]. As the differences between CH and CRF have not been fully understood, these findings indicated that further research is needed to investigate precisely what biomechanical properties are represented by CH and CRF. In the current study, CH was significantly decreased by increasing degree of myopia without significant changes of CRF. Our results indicated that the myopia-related structural change of CH may be independent of CRF.

Age-related changes of the corneal structure have been observed in previous histological studies [43, 44]. It has been reported that interfibrillar spacing reduced with age and collagen fibril cross-linking increased with age [44]. This might result in changes of cornea biomechanical properties. By using ORA, the relationship between age and* in vivo* corneal biomechanical properties has been investigated [18–20, 45–47]. Most studies reported that age was significantly associated with CH and CRF. Kamiya et al. [45] reported that age was negatively and significantly correlated with CH in 204 normal eyes of 204 healthy Japanese volunteers (age range, 19 to 89 years). In another study, Kotecha et al. [46] also found that both CH and CRF were negatively associated with age. In contrast, we did not detect a significant correlation between CH, CRF, and age in our study population. One possible explanation is that the age range in our study is relatively narrow. Of note, most of the subjects included in our study are young myopic subjects (95% confidence intervals for age: 22.35–23.69 years).

In conclusion, both CH and CRF were positively correlated with CCT. Lower CH but not CRF was associated with increasing degree of myopia. Eyes with a longer axial length may have a compromised corneal mechanical strength. A clinical assessment of cornea biomechanical properties should be interpreted in the context of CCT and the myopic status.

## Figures and Tables

**Table 1:** Corneal biomechanical characteristics in the study population (*n* = 135).

	Mean ± SD	Range
CRF (mmHg)	9.64 ± 1.57	5.82 to 13.89
CH (mmHg)	9.82 ± 1.34	6.85 to 13.24
IOPg (mmHg)	14.56 ± 2.89	5.90 to 20.20
IOPcc (mmHg)	15.80 ± 2.60	6.20 to 21.20
CCT (*μ*m)	547.7 ± 27.3	488.0 to 610.0
Cornea curvature (mm)	7.78 ± 0.23	6.86 to 8.54

CH: cornea hysteresis, CRF: corneal resistance factor, IOPcc: cornea-compensated intraocular pressure, IOPg: Goldmann-correlated intraocular pressure, CCT: central corneal thickness, and SD: standard deviation.

**Table 2:** Correlation analysis between axial length, spherical equivalent, age, central corneal thickness (CCT), cornea curvature, IOPcc, and corneal biomechanics (Pearson correlation analysis, *n* = 135).

	CH		CRF	
	*r*	*p*	*r*	*p*
Age	0.024	0.781	−0.035	0.690
Spherical equivalent	0.234	0.006	0.149	0.140
Axial length	−0.269	0.002	0.135	0.118
CCT	0.454	<0.001	0.521	<0.001
IOPcc	−0.357	<0.001	0.203	0.018
Cornea curvature	−0.137	0.168	−0.049	0.627

CH: cornea hysteresis, CRF: corneal resistance factor, IOPcc: cornea-compensated intraocular pressure, and CCT: central corneal thickness.

**Table 3:** Multivariate regression analysis with the corneal hysteresis (CH) as the dependent variable and axial length, central corneal thickness (CCT), and IOPcc as the independent variables (*n* = 135).

	Unstandardized coefficient	Standardized coefficient	*p*
Axial length	−0.230	−0.178	0.014
CCT	0.024	0.472	<0.001
IOPcc	−0.185	−0.355	<0.001
*R* ^2^ = 0.392, *p* < 0.001			

CH: cornea hysteresis, IOPcc: cornea-compensated intraocular pressure, and CCT: central corneal thickness.

**Table 4:** Multivariate regression analysis with the corneal resistance factor (CRF) as the dependent variable and central corneal thickness (CCT) and IOPcc as the independent variables (*n* = 135).

	Unstandardized coefficient	Standardized coefficient	*p*
CCT	0.030	0.510	<0.001
IOPcc	0.094	0.155	0.039
*R* ^2^ = 0.295, *p* < 0.001			

CRF: corneal resistance factor, IOPcc: cornea-compensated intraocular pressure, and CCT: central corneal thickness.
